# *Cis*-Acting Relaxases Guarantee Independent Mobilization of MOB_Q__4_ Plasmids

**DOI:** 10.3389/fmicb.2019.02557

**Published:** 2019-11-08

**Authors:** M. Pilar Garcillán-Barcia, Raquel Cuartas-Lanza, Ana Cuevas, Fernando de la Cruz

**Affiliations:** Instituto de Biomedicina y Biotecnología de Cantabria (Universidad de Cantabria – Consejo Superior de Investigaciones Científicas), Santander, Spain

**Keywords:** mobilizable plasmids, horizontal gene transfer, MOB_Q_ relaxase, *cis-*acting relaxase, plasmid coexistence, bacterial conjugation

## Abstract

Plasmids are key vehicles of horizontal gene transfer and contribute greatly to bacterial genome plasticity. In this work, we studied a group of plasmids from enterobacteria that encode phylogenetically related mobilization functions that populate the previously non-described MOB_Q__4_ relaxase family. These plasmids encode two transfer genes: *mobA* coding for the MOB_Q__4_ relaxase; and *mobC*, which is non-essential but enhances the plasmid mobilization frequency. The origin of transfer is located between these two divergently transcribed *mob* genes. We found that MPF_I_ conjugative plasmids were the most efficient helpers for MOB_Q__4_ conjugative dissemination among clinically relevant enterobacteria. While highly similar in their mobilization module, two sub-groups with unrelated replicons (Rep_3 and ColE2) can be distinguished in this plasmid family. These subgroups can stably coexist (are compatible) and transfer independently, despite origin-of-transfer cross-recognition by their relaxases. Specific discrimination among their highly similar *oriT* sequences is guaranteed by the preferential *cis* activity of the MOB_Q__4_ relaxases. Such a strategy would be biologically relevant in a scenario of co-residence of non-divergent elements to favor self-dissemination.

## Introduction

Mobilizable plasmids are small genetic elements transmissible by conjugation with the assistance of a helper conjugative plasmid. They encode a relaxase, and usually a relaxase accessory protein (RAP), which are in charge of the conjugative DNA processing at a specific site of the origin of transfer (*oriT*) called *nic*. Mobilizable plasmids lack the transfer genes required for establishing a conjugative bridge (mating pair formation system, MPF) to the recipient cell, as well as the type IV coupling protein (T4CP) that puts in contact relaxosome and MPF and thus depend on conjugative plasmids to be transferred (Garcillán-Barcia and de la Cruz, 2013).

According to their relaxase, transmissible plasmids were phylogenetically classified into MOB families (Francia et al., 2004; Garcillán-Barcia et al., 2009). Currently, nine relaxase MOB classes are defined, and five of them (MOB_P_, MOB_F_, MOB_Q_, MOB_H_, and MOB_C_) are prevalent in transmissible plasmids hosted in γ-Proteobacteria. Plasmids gathered in a relaxase MOB family share similar genomic traits. Relaxase MOB classification has thus shown to be a good predictor of the plasmid backbone (Garcillán-Barcia and de la Cruz, 2013; Fernandez-Lopez et al., 2017). Mobilizable plasmids resident in γ-Proteobacteria form phylogenetically related clusters mainly within two relaxase MOB classes: MOB_P_ and MOB_Q_ (Garcillán-Barcia et al., 2009). Relevant examples are ColE1-like plasmids, grouped in family MOB_P__5_; IncQ1 plasmids, such as RSF1010/R1162, gathered in MOB_Q__11_; and IncQ2 plasmids, such as pTC-F14, in family MOB_P__14_ (Garcillán-Barcia et al., 2009; Garcillán-Barcia and de la Cruz, 2013). An additional clade of small plasmids encoding MOB_Q_ relaxases, previously classified as MOB_Qu_, and here redefined as MOB_Q__4_, was observed in a phylogenetic reconstruction of this relaxase family (Garcillán-Barcia et al., 2009).

A pair of degenerate primers specific for MOB_Q__4_ plasmids was implemented in the Degenerate PCR MOB Typing (DPMT) approach developed by Alvarado et al. (2012) to detect and classify transmissible plasmids. This method revealed the abundance of MOB_Q__4_ plasmids in clinical isolates of enterobacteria (Alvarado et al., 2012; Garcillán-Barcia et al., 2015), previously unnoticed by other plasmid typing methods. Whole-genome sequencing of clinical *E. coli* isolates also uncovered the presence of this kind of plasmids (Brolund et al., 2013; de Toro et al., 2014; Lanza et al., 2014). Prototype plasmids pIGWZ12 and ColE9-J (ColE2-like) cluster within the MOB_Q__4_ clade. They are stable, theta-replicating, high copy-number, narrow host-range plasmids, whose replication systems have been extensively studied (Yasueda et al., 1989, 1994; Yagura et al., 2006; Zaleski et al., 2006, 2015). Here, we uncovered the diversity of MOB_Q__4_ plasmids, determined the helper conjugative plasmids responsible for their dissemination, and established their behavior in terms of stability and transfer.

## Materials and Methods

### Plasmid Construction

MOB_Q__4_ plasmid derivatives were constructed by isothermal assembly of linear DNA fragments from PCR reactions, following the Gibson method (Gibson et al., 2009, 2015). The MOB_Q__41_ backbone (replication and mobilization regions), based on the complete sequence of the pE2022_4 plasmid [GenBank Acc. No. KT693143 (Lanza et al., 2014)], was linked to a kanamycin-resistance gene [coordinates 272 to 1216 of pSEVA211, GenBank Acc. No. JX560326 (Silva-Rocha et al., 2013)] and a cerulean fluorescent protein gene [coordinates 41 to 1091 of pNS2-φVL (Dunlop et al., 2008)], generating plasmid pRC1. The MOB_Q__42_ backbone (replication and mobilization regions) was obtained by PCR amplification from the *E. coli* isolate HUMV 04/979 (Garcillán-Barcia et al., 2015), which contains a ColE9-J-like plasmid (coordinates 5102 to 7577, GenBank Acc. No. NC_011977.1). It was joined to a chloramphenicol resistance gene (coordinates 272–1072 of pSEVA311, GenBank Acc. No. JX560331 (Silva-Rocha et al., 2013)] and mCherry fluorescent protein gene (*cfp*, coordinates 1092–2117 of pNS2-φVL (Dunlop et al., 2008)], generating plasmid pRC2. MOB_Q__4_ plasmids lacking the *mobC* ORF (from start to stop codon) were constructed by self-ligation of a single PCR fragment from either pRC1 or pRC2, producing plasmids pRC3 and pRC4, respectively.

**FIGURE 1:**
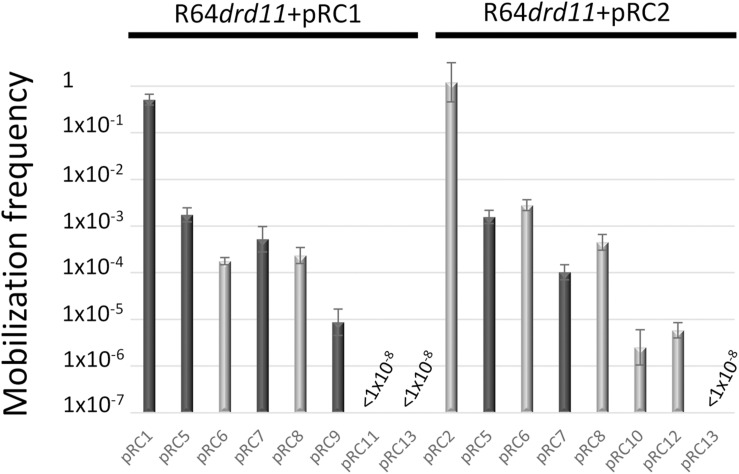
Schematic representation of the MOB_Q__4_ DNA segments included in a series of recombinant plasmids. The mobilization region of MOB_Q__4_ plasmids includes *mobC* and *mobA* genes, represented by large, horizontal gray arrows. The extent of the mobilization region included in each construction is represented by a gray bar. The plasmid names for the MOB_Q__41_–based constructions are listed in the left column, while those for MOB_Q__42_-based constructions are in the right column. Plasmids pRC1, pRC2, pRC3, and pRC4 also include the replication module of MOB_Q__41_ or MOB_Q__42_ plasmids.

Additional plasmids were constructed to delimit the *oriT* region. A schematic representation of the fragments included in each construction is depicted in Figure 1. Such fragments were individually assembled to coordinates 1–1030 and 1360–3001 of vector pSEVA631 (GenBank Acc. No. JX560348). Plasmids pRC5 and pRC6 contained a fragment including the *mobC* gene, the 178bp intergenic region between *mobC* and *mobA* and the first 400 nucleotides of the *mobA* gene from pRC1 and pRC2, respectively. Plasmids pRC7 and pRC8 included only the 178bp intergenic fragment (Supplementary Figure S1), located between genes *mobA* and *mobC* of pRC1 and pRC2, respectively. Plasmids pRC14 and pRC15 contain the *oriT* regions of pRC7 and pRC8 but cloned in the inverse orientation. Plasmids pRC11 and pRC9, respectively included portions 1–70 and 71–178 of the intergenic fragment between genes *mobA* and *mobC* of pRC1, while the same portions from pRC2 were included in pRC12 and pRC10, respectively. A pSEVA631 fragment containing coordinates 1–1030 and 1360–3001 was self-ligated, generating the non-mobilizable vector pRC13, which was used as a control in the mating experiments.

### Stability Assays

Plasmids pRC1 and pRC2 were introduced in the *recA*^+^ and *recA*^–^ isogenic strains UB1636 (F^–^ *lys his trp rpsL*) (Achtman et al., 1971) and UB1637 (F^–^ *lys his trp rpsL recA56*) (de la Cruz and Grinsted, 1982), either independently to check for their stability or both together to check for their compatibility. Single colonies were inoculated in Lysogeny-Broth (LB) supplemented with kanamycin at 50 μg/ml (for pRC1-containing strains) or chloramphenicol at 25 μg/ml (for pRC2-containing strains) and grown to saturation at 37°C with agitation (150 rpm). A volume of 9.7 μl was transferred from saturated cultures to 10 mL of fresh LB media without antibiotics and grown to saturation in the same conditions. Rounds of transfer and growth were repeated up to 80 generations. The proportion of plasmid-bearing cells in the population was monitored by replica-plating 100 colonies in LB-agar supplemented with the appropriate antibiotics every 10 generations. A larger number of cells was inspected by fluorescence microscopy and, in the case of pRC1-containing cells, also by flow cytometry. Live cells were visualized using a Leica AF6500 microscope at 63x magnification. CFP and mCherry signals were monitored using BP filters (Excitation 434/17 – Emission 479/40 for CFP, Excitation 562/40 – Emission 641/75 for mCherry). Images were obtained using an iXon885 EM CCD Camera (Andor) and up to 1000 cells were analyzed in each case. Fluorescence emission was measured by flow cytometry using a FACS Canto II flow cytometer (Becton Dickinson) equipped with a 488 nm solid state laser for excitation. The cyan fluorescence of 20,000 events was detected using a 525/20 filter.

### Mating Assays

Conjugative plasmids used in this work are listed in Supplementary Table S1. They were tested as helpers of the MOB_Q__4_ plasmids in surface mating experiments, following the procedure described by del Campo et al. (2012). *E. coli* strain DH5α (F^–^ *endA1 glnV44 thi-1 recA1 relA1 gyrA96 deoR nupG purB20* φ80d*lacZ*ΔM15 Δ(*lacZYA-argF*)U169, hsdR17(*rK*^–^*mK*^+^(), λ^–^) (Grant et al., 1990) containing different plasmid combinations was used as donor and BW25113 (*lacI*^q^ *rrnB*T14 Δ*lacZ*WJ16 *hsdR514* Δ*araBAD*AH33 Δ*rhaBAD*LD78), BW25993 (*lacI*^q^ *hsdR514* Δ*araBAD*AH33 Δ*rhaBAD*LD78) (Datsenko and Wanner, 2000) as recipient. Donor and recipient strains were mixed in a 1:1 ratio, deposited onto an LB-agar surface and incubated for 1 h at 37°C (except when drR27 was used as a helper, in which case matings were carried out at 25°C). Then, the mixture was resuspended in LB and plated in the presence of appropriate antibiotics. Conjugation frequencies were expressed as the number of transconjugants per donor cell.

### Phylogenetic Analysis

The 300 N-terminal residues of the MobA relaxase of plasmid ColE9-J were used as a query in a BLASTP search (Altschul et al., 1997) (*e*-value: 1xE-3). The homologous sequences were aligned using MUSCLE (Edgar, 2004). TrimAl v1.4 was used to calculate the average identity between sequences in the alignment (Capella-Gutiérrez et al., 2009). ProtTest 3 was used to estimate the best model of protein evolution for our set (Guindon and Gascuel, 2003; Darriba et al., 2011). RAxML version 7.2.7 (Stamatakis, 2006) was used for phylogenetic reconstruction. Using the JTTGAMMA model 10 maximum likelihood (ML) searches trees were inferred and support values were assigned to each node of the best tree from 1000 bootstrap searches. Relaxase of the pXF5847 plasmid (GenBank Acc. no. YP_009076807.1) was used as outgroup.

### 3D Structure Prediction

Phyre2 was used to predict the 3D structure of the MobA relaxase domains of plasmids pE2022_4 and ColE9-J (Kelley et al., 2015), which were visualized using PyMOL (Schrödinger, 2015).

## Results and Discussion

### Analysis of MOB_Q__4_ Plasmids

MOB_Q_ is a broad relaxase class that encompasses several families, each of which includes related plasmid backbones: MOB_Q__1_ comprises relaxases of mobilizable broad host-range IncQ1-like plasmids; MOB_Q__2_, conjugative relaxases of pTi and many rhizobial plasmids; MOB_Q__3_, conjugative broad host-range plasmids resident in gram-positive, such as pIP501 (Garcillán-Barcia et al., 2009). In this previous study, many MOB_Q_ plasmids were not ascribed to a specific subclassification due to either low resolution of the clades or lack of information on the plasmid members. Here, we focused on one of these poorly defined clades, now named MOB_Q__4_, prompted by the fact that these relaxases have been recurrently detected in enterobacterial clinical isolates (Alvarado et al., 2012; Brolund et al., 2013; de Toro et al., 2014; Lanza et al., 2014; Garcillán-Barcia et al., 2015).

**FIGURE 2:**
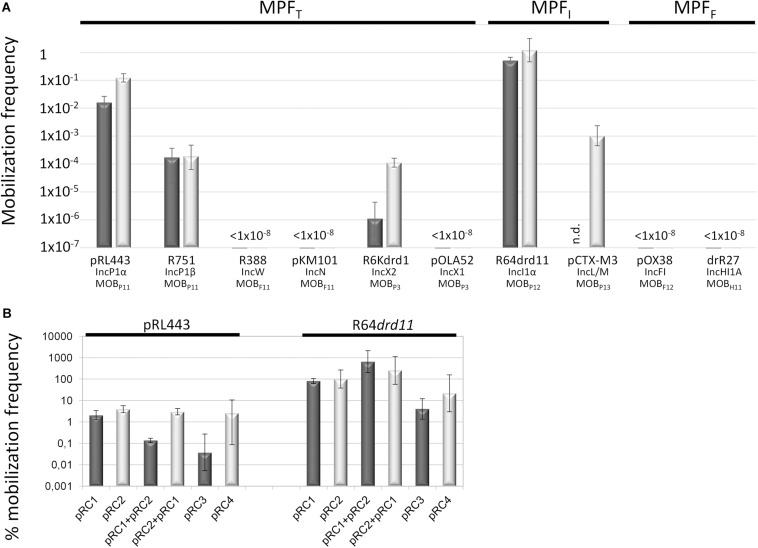
The MOB_Q__4_ relaxase family. **(A)** Maximum-likelihood phylogenetic reconstruction of the N-terminal domain of MOB_Q__4_ relaxases is shown. MobA relaxase of plasmid pXF5843 was used as outgroup. Bootstrap values of relevant nodes are indicated. Families Q41 and Q42 are shadowed in dark and light gray, respectively. A prototype backbone of each MOB_Q__4_ group is represented to the right of the corresponding clade. Genes of the mobilization module are represented in the same gray color pattern. The elements of the replication module are dotted (for MOB_Q__41_) or striped (for MOB_Q__42_). Genes of the colicin operon and the hypothetical proteins are depicted in a white background. **(B)** Multiple alignment of the three conserved MOB_Q_ relaxase motifs (Garcillán-Barcia et al., 2009). (1) MOB_Q__41_ relaxases, with the exception of pMG828-2; (2) pMG828-2; (3) MOB_Q__42_ relaxases. Putative catalytic residues are indicated by black triangles over the amino acid sequences. **(C)** Protein 3D-structure superposition of MOB_Q__41_, MOB_Q__42_ and MOB_Q__11_ relaxases. In gray, the minimal relaxase domain of MobA of plasmid R1162/RSF1010 used as a model (PDB 2NS6); in blue and magenta the predicted structures of the homologous domains of the MOB_Q__41_ (MobA of pE2022_4) and MOB_Q__42_ (MobA of ColE9-J) relaxases, respectively. Key residues of MOB_Q__4_ relaxases are highlighted as sticks.

The phylogenetic reconstruction, based on the first N-terminal 300 residues of MOB_Q__4_ relaxases produced two clusters, MOB_Q__41_ and MOB_Q__42_ (Figure 2A and Supplementary Table S2). This relaxase domain contains the three relaxase motifs (Figure 2B) and share 84% average amino acid identity (97 and 90% for individual MOB_Q__41_ and MOB_Q__42_ groups, respectively). The 3D structure prediction of the relaxase domain of MOB_Q__41_ and MOB_Q__42_ plasmids rendered MOB_Q_ relaxases NES [plasmid pLW1043, PDB Acc. No. 4HT4 (Edwards et al., 2013)] and MobA [plasmid R1162/RSF1010, PDB Acc. No. 2NS6, (Monzingo et al., 2007)] as best hits (100% confidence). The superimposed structures pointed to MOB_Q__4_ amino acids Y25 (motif I), E87 and E89 (motif II), and H125, H133 and H135 (motif III) as homologs of the MobA_R1162 catalytic residues Y25, E74 and E76, and H112, H120 and H122, respectively (Figure 2C). Contrary to the high conservation of the N-terminal domain among members of both MOB_Q__4_ subgroups, the amino acid identity of the C-terminal part of the MOB_Q__4_ relaxases dropped to 35%. This C-terminal domain exhibited low homology to SogL primases of IncI1 plasmids.

Each MOB_Q__4_ subclade groups highly related backbones (Figure 2A). MOB_Q__41_ are cryptic, small-size plasmids (Supplementary Table S2). Their backbone contains only four genes encoding a replication initiation protein (Rep), a relaxase (MobA), a putative relaxase accessory protein (MobC) and a hypothetical protein. The genes for the last two are generally not annotated. Besides the above-mentioned replication and mobilization genes, MOB_Q__42_ plasmids also contain a colicin operon, including colicin, immunity and lysis genes, following the synteny of Group A nuclease colicins (Cascales et al., 2007). Plasmids ColE9-J and pO111_4 contain a second, partial colicin operon.

The MOB_Q__4_ subdivision in two relaxase groups matches with the presence of two different replicons (Supplementary Table S2) and this family thus encompasses at least two plasmid species as defined by Fernandez-Lopez et al. (2017). MOB_Q__41_ plasmids encode a replication initiation protein that belongs to the Rep_3 superfamily [PF01051 in the Pfam classification (Finn et al., 2016)], with no defined group in the PlasmidFinder classification (Carattoli et al., 2014). MOB_Q__42_ plasmids encode ColE2-like initiators (Pfam PF03090 + PF08708), classified as Col156 by PlasmidFinder. Plasmids pIGWZ12 and ColE9-J exemplify each cluster. They are stable, theta-replicating, high copy number plasmids (15 and 10 copies per chromosome molecule, respectively (Takechi et al., 1994; Zaleski et al., 2012). The origin of replication of plasmid pIGWZ12 was located upstream the *rep* gene. It contains iterons, an A+T rich region and four DnaA boxes (Zaleski et al., 2006, 2015). The iterons were found to be the incompatibility determinants (Zaleski et al., 2015). ColE2-like plasmids, such as ColE9-J, form a group of closely related elements that share an identical priming mechanism, mediated by the plasmid-encoded Rep protein (Horii and Itoh, 1988; Itoh and Horii, 1989; Yasueda et al., 1989; Hiraga et al., 1994). The origin of replication consists of 32 bp located downstream of the *rep* gene, containing two directly repeated sequences (Kido et al., 1991; Nomura et al., 1991; Yagura and Itoh, 2006; Yagura et al., 2006). In ColE2-like plasmids, the *rep* gene expression is post-transcriptionally controlled by a plasmid-encoded RNA (*RNAI*), which binds the untranslated 5′ region of the *rep* mRNA, preventing its translation (Sugiyama and Itoh, 1993; Takechi et al., 1994; Yasueda et al., 1994). MOB_Q__42_ plasmids contain a *cer*-like site (Hiraga et al., 1994), an indication that they use a host site-specific recombination system for resolving multimers to monomers as ColE1-like plasmids do (Summers and Sherratt, 1984, 1988; Summers, 1998).

All completely sequenced MOB_Q__4_ plasmids come from hosts of the Enterobacteriaceae family (Supplementary Table S2). They were isolated from different backgrounds: *Salmonella enterica* isolated from pork meat (pSD4.0) (Bleicher et al., 2013), pork feces (p4_TW-Stm6) (Dyall-Smith et al., 2017) and human systemic infection (pYU39_5.1) (Calva et al., 2015), multidrug-resistant environmental *E. coli* (pSMS35_4) (Fricke et al., 2008), commensal *E. coli* (pSE11-6) (Oshima et al., 2008), enterohemorrhagic *E. coli* strains of the O26 and O111 serogroups (pO26-S4 and pO111_4) (Ogura et al., 2009; Fratamico et al., 2011), extended-spectrum beta-lactamase producing *E. coli* clinical isolates (pE2022_4, pFV9873_1, pEC147-3 and pEC08-6) (Brolund et al., 2013; Lanza et al., 2014), *E. coli* isolated from human urinary tract (pVR50F) (Beatson et al., 2015) and bloodstream infections (pSF-468-4) (Stephens et al., 2015), as well as porcine extraintestinal pathogenic *E. coli* strain (p2PCN033) (Liu et al., 2015), among others (Supplementary Table S2). None of these plasmids contain antibiotic-resistance genes. There is still no clue on the selective advantage provided by the cryptic MOB_Q__41_ plasmids. In the case of MOB_Q__42_ plasmids, the fact that all carry colicin operons, *a priori* an advantageous trait for the bacterial host, could explain the abundance of this type of plasmids. For example, the MOB_Q__42_ plasmid pDPT1 was stably acquired by a Vietnamese *Shigella sonnei* strain in the mid-1990s, and became fixed in the evolving bacterial population (Holt et al., 2013). The colicin E5 produced by pDPT1 was highly bactericidal against non-immune *Shigella* and *E. coli* strains. The acquisition of the pDPT1 colicin plasmid, coinciding with the high increase of dysentery produced by this strain, suggests that pDPT1 conferred a beneficial function to its host (Holt et al., 2013).

### Stability and Co-residence of MOB_Q__4_ Plasmids

To study the MOB_Q__4_ plasmids, two derivatives were constructed, pRC1 and pRC2. They included the replication and mobilization modules of the MOB_Q__41_ and MOB_Q__42_ backbones, respectively. Antibiotic-resistance and fluorescent protein genes were also included as reporters. Plasmid stability and compatibility were assayed in *recA*^+^ and *recA*^–^ *E. coli* strains by propagating the plasmids either alone or in combination during 80 generations. Despite the cargoes loaded in plasmids pRC1 and pRC2, the percentage of plasmid retention in the bacterial population was 100%, suggesting that the MOB_Q__4_ backbone confers a minimized fitness cost to its enterobacterial host (San Millan and MacLean, 2017). Besides stability in *E. coli*, both MOB_Q__4_ plasmid species also exhibited full compatibility (100% retention of both after 100 generations), as could be expected due to their different replicons (Novick, 1987), and ruling out other plasmid-encoded traits out of the replication module that could interfere with the stable vertical inheritance of each other.

### Mobilization of MOB_Q__4_ Plasmids by Different MPF Systems

Since mobilizable plasmids do not encode the mating pair formation system neither the T4CP, their transfer relies on auto-transmissible plasmids. We wondered which conjugative plasmids could be responsible for the dissemination of the MOB_Q__4_ plasmids. Not all conjugative plasmids are equally efficient at supplying these functions to a specific mobilizable plasmid (Cabezón et al., 1994, 1997). The contacts established between the relaxosome of the mobilizable plasmid and the T4CP-MPF of the helper plasmid are crucial in the transfer process. ColE1-like MOB_P__5_ plasmids are efficiently mobilized by IncF-MOB_F__12_ (e.g., F) and IncI1-MOB_P__12_ (e.g., R64*drd11*) plasmids (Cabezón et al., 1997). IncQ1-MOB_Q__1_ plasmids, such as RSF1010, are transferred by IncP1-MOB_P__11_ helper plasmids (e.g., RP4) (Cabezón et al., 1997; Meyer, 2009). pMV158-like plasmids (MOB_V__1_) are mobilized by IncP1-MOB_P__11_ and Inc18-MOB_Q__3_ (e.g., pIP501) plasmids (Lorenzo-Díaz et al., 2014).

We looked for reports providing indirect evidence on MOB_Q__4_ plasmid mobilization through conjugation. In a survey for the presence of transmissible plasmids in a multidrug *E. coli* collection, MOB_Q__4_ transconjugants were obtained from seven out of the eight MOB_Q__4_ containing clinical isolates (Garcillán-Barcia et al., 2015). In all cases, a MOB_P__12_-MPF_I_ plasmid, presumptively the helper, was also present in both, donor and transconjugant cells. Similarly, the MOB_Q__41_ plasmid pSD4.0 and the IncI1 plasmid pSD107 were found in *E. coli* transconjugants arisen from a mating with *Salmonella enterica* (Bleicher et al., 2013).

**FIGURE 3:**
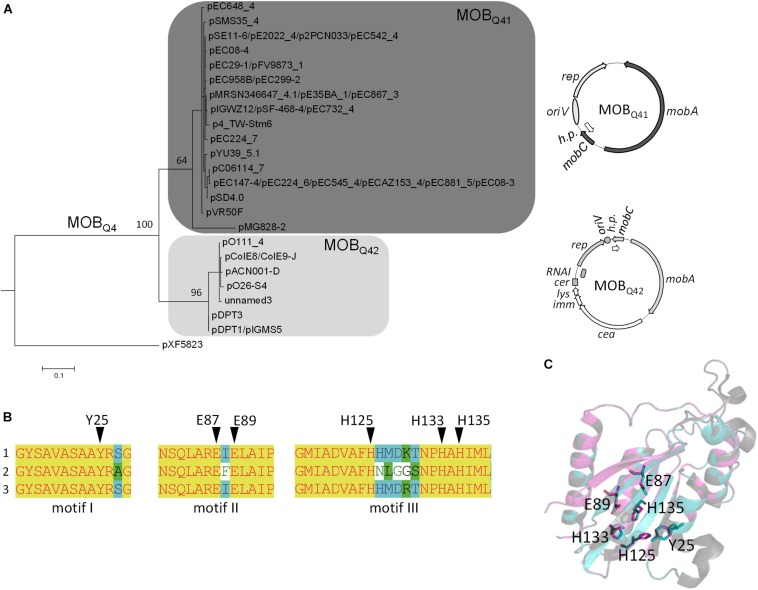
Mobilization frequencies of MOB_Q__4_ plasmids by a series of helper plasmids. The mobilization frequency was calculated as the number of transconjugants containing the MOB_Q__4_ plasmid per donor cell. Figures are the average of at least six independent experiments. **(A)** Dark- and light-gray bars indicate the mobilization frequencies of pRC1 (MOB_Q__41_) and pRC2 (MOB_Q__42_) plasmids, respectively. Below the bars the helper plasmid used in each case, as well as its corresponding Inc and MOB groups, are indicated. The MPF types of the helper plasmids are indicated in the upper part of the figure. **(B)** The mobilization efficiencies of the MOB_Q__4_ plasmids are relativized to the helper plasmid transfer rates (100%). The mobilization efficiencies of MOB_Q__41_ and MOB_Q__42_-based constructions are represented by dark- and light-gray bars, respectively. pRC1 + pRC2 indicates the mobilization frequency of pRC1 when coresident with pRC2. pRC2 + pRC1 indicates the mobilization frequency of pRC2 when coresident with pRC1.

Three conjugative MPF types (MPF_T_, MPF_F_, and MPF_I_) are prevalent in Enterobacteriaceae (Smillie et al., 2010; Guglielmini et al., 2014), the taxonomic family where MOB_Q__4_ plasmids have been found. In this study, a set of conjugative plasmids representative of these MPF families were tested as helpers for the mobilization of MOB_Q__4_ plasmids (Supplementary Table S1). Not all of them were equally efficient (Figures 3A,B and Supplementary Table S3). R64*drd11*, the prototype of IncI1α-MOB_P__12_ plasmids, which encodes a MPF_I_ conjugative apparatus, was the most efficient helper. Another MPF_I_ plasmid, pCTX-M3 (IncL/M-MOB_P__13_), was also an efficient helper. Co-residence with MPF_I_ plasmids has been reported for the MOB_Q__4_ plasmids pSE11-6 (Oshima et al., 2008), pSD4.0 (Bleicher et al., 2013), pEC147-4 (Brolund et al., 2013), pO26-S4 (Fratamico et al., 2011), pDPT1 (Holt et al., 2013), and pE2022_4 (Lanza et al., 2014).

On the other hand, MPF_F_-type plasmids [e.g., IncF-MOB_F__12_ (F) or IncHI1-MOB_H__11_ (R27) plasmids], which show high prevalence in enterobacteria, were not appropriate for MOB_Q__4_ mobilization. MPF_T_ plasmids behaved unevenly as MOB_Q__4_ mobilizers. IncP1-MOB_P__11_ (RP4 and R751) and IncX2-MOB_P__3_ (R6K*drd1*) plasmids rendered MOB_Q__4_ transconjugants, while IncW-MOB_F__11_ (R388), IncN-MOB_F__11_ (pKM101) or IncX1-MOB_P__3_ (pOLA52) did not. Contrary to IncP, IncW and IncN plasmids, most IncF, IncI1, IncH, and IncX plasmids are naturally repressed for conjugation. In this study, we used derepressed variants of IncF (pOX38 and R100-1), IncI1α (R64*drd11*), IncHI1 (drR27), and IncX2 (R6K*drd1*) plasmids, but not a derepressed IncX1. IncX1 and IncX2 plasmids are highly similar in their conjugation genes. Taking into account that the IncX2 derepressed plasmid R6K*drd1* was not efficient at mobilizing MOB_Q__4_ plasmids (Figure 3A and Supplementary Table S3), and that the IncX1 plasmid pOLA52 self-transfers at low frequency (around 10^–4^ per donor) (Sørensen et al., 2003), the lack of mobilization of the MOB_Q__4_ plasmids pRC1 and pRC2 by pOLA52 is not surprising. The widely different mobilization efficiencies displayed by the two IncP1-MOB_P__11_ helpers used is more curious. RP4 and R751 are prototypes of the α and β divisions of the IncP1 backbones, respectively. Despite the high conservation of their transfer genes, the kanamycin-sensitive RP4 derivative, pRL443, was 100–1000 times more efficient than R751 as a MOB_Q__4_ helper. Noticeable differences were also observed for these two conjugative plasmids at transferring IncQ2-MOB_P__14_ mobilizable plasmids pTC-F14 and pTF-FC2 (van Zyl et al., 2003). The common characteristic of the MOB_Q__4_ mobilizers was their belonging to the MOB_P_ relaxase class. This could indicate that the MOB_Q__4_ relaxosomes interact more efficiently with the T4 encoded by these MOB_P_ plasmids.

### Effect of Co-residence in the MOB_Q__4_ Plasmid Mobilization

Bacterial co-infection with multiple plasmids is common in nature (San Millan et al., 2014). Co-residence of compatible plasmids may lead to intracellular interactions that negatively or positively affect plasmid transfer rates (Gama et al., 2017a,b,c; Getino et al., 2017). Among them, plasmid-encoded fertility inhibition systems that block transmission of unrelated plasmids from the same donor cell have been intensively studied (Maindola et al., 2014; Gama et al., 2018; Getino and de la Cruz, 2018). Besides, competition of two relaxosomes for the same T4CP-MPF can result in the preponderance of one them (Cascales et al., 2005), a fact relevant for any mobilizable plasmid. Cohabitation of two or more mobilizable plasmids that use the same mating apparatus could affect each other’s transfer. To test whether the mobilization of the MOB_Q__41_ plasmid was affected by co-residence with a MOB_Q__42_ plasmid and vice versa, pRC1 and pRC2 were introduced conjointly with the helper plasmid (either pRL443 or R64*drd11*) in the same cell (Figure 3B). Curiously, presence of pRC1 did not produce a significant variation in pRC2 transfer. In turn, pRC2 produced one-log decrease in pRC1 transfer by pRL443. However, this moderate negative effect was not exhibited when using R64*drd11* as a helper: on the contrary, pRC2 presence resulted in one-log increase in pRC1 transfer. Testing different combinations of MOB_Q__41_, MOB_Q__42_ and helpers would be necessary to deeper assess the impact of residing together in MOB_Q__4_ horizontal propagation.

### *mobC* Deletion Effect in the Mobilization Efficiency

Many conjugative and mobilizable plasmids encode RAPs that recognize and bind their cognate *oriT* sequence probably favoring a single-stranded state around the *nic* site (de la Cruz et al., 2010). Deletion of RAP genes *trwA* of R388 (Moncalián et al., 1997), *nikA* of R64 (Furuya et al., 1991), *mobB* and *mobC* of plasmids pTC-F14 and pTF-FC2 (van Zyl et al., 2003), *traJ* and *traK* of RP4 (Guiney et al., 1989), *mobC* of R1162/RSF1010 (Brasch and Meyer, 1986), and *mbeC* of ColE1 (Varsaki et al., 2009) resulted in drastic decrease of plasmid transfer. All MOB_Q__4_ plasmids encode a gene, called *mobC*, which is located adjacent to *oriT* and transcribed opposite to the *mobA* relaxase gene (Figure 1). Most of the *mobC* genes are not annotated, so we updated their annotation, as listed in Supplementary Table S2. The MobC proteins of MOB_Q__4_ plasmids are small (less than 100 amino acids) and showed no homology to other RAPs (by using PSI-Blast). To check whether MobC plays a role in the MOB_Q__4_ plasmid mobilization, *mobC* deletion mutants were constructed from pRC1 and pRC2, respectively producing pRC3 and pRC4 (Figure 1). A moderate decrease in mobilization was observed in the *mobC*^–^ variants: 1.5-log reduction for pRC3 and 0.6-log for pRC4, when using R64*drd11* as a helper (Figure 3B). MobC is thus not absolutely essential for MOB_Q__4_ plasmid mobilization. This is an interesting difference to other plasmid groups, which should be further investigated. It is conceivable that some MOB_Q__4_ plasmids can be found, the mobilization of which is independent of RAPs.

### *In trans* Mobilization of *oriT*_MOB_Q__4_-Containing Vectors

**FIGURE 4:**
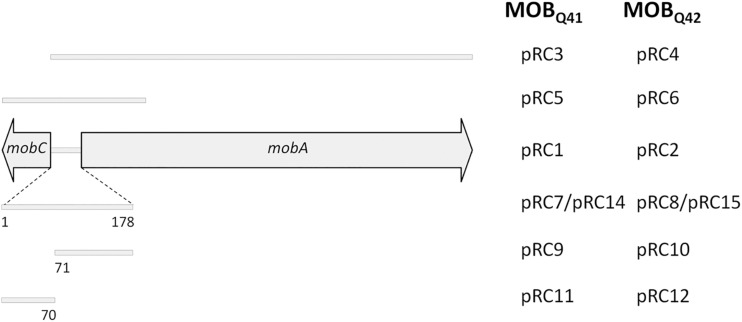
*In trans* mobilization of *oriT* fragments. R64*drd11*-mediated mobilization frequencies of pRC1 and plasmids containing fragments of its *oriT* are represented by dark-gray bars, while those of pRC2 and its derivatives are in light-gray bars. The bars represent the average of at least six experiments.

The 178 bp intergenic region comprised between the *mobC* and *mobA* genes of MOB_Q__4_ plasmids was assembled with an *oriT*-lacking fragment of vector pSEVA631. The resulting constructions, pRC7 (for MOB_Q__41_) and pRC8 (for MOB_Q__42_) (Figure 1), were introduced in donor strains to check for their mobilization. The transfer proteins were supplied *in trans*: the corresponding mobilizable plasmid (pRC1 or pRC2) provided the relaxosomal proteins, while the conjugative plasmid (R64*drd11*) supplied the T4CP and MPF. Plasmids pRC7 and pRC8 were transferred to the recipient population, but 1000-fold less efficiently than their corresponding *mobA*^+^*mobC*^+^ partners (pRC1 and pRC2) (Figure 4). This result was confirmed by using plasmids pRC14 and pRC15, instead of pRC7 and pRC8, in the mobilization experiments. Plasmids pRC14 and pRC15 contained the same *oriT* region present in pRC7 and pRC8, but cloned in the inverse orientation. Besides, to avoid losing any *oriT*-related function, larger segments including also the *mobC* gene and the first 431 bp of the *mobA* gene [pRC5 and pRC6 (Figure 1)], were analyzed. Here again relaxase, T4CP and MPF components were provided *in trans*. Plasmids pRC5 and pRC6 behave similarly to pRC7 and pRC8, and were mobilized at least 500-fold less than pRC1 and pRC2 (Figure 4).

MOB_Q__4_ relaxases showed thus a *cis*-acting preference for their *oriT*s, performing at least 500-fold better on a *cis* than on a *trans oriT* substrate. The *cis-*acting preference is a characteristic exhibited by some DNA-binding proteins, such as the TnpA transposases of Tn*10*, Tn*5* and Tn*903* (Morisato et al., 1983; Derbyshire et al., 1990; DeLong and Syvanen, 1991). Relaxases generally lack a *cis* preference for their *oriT*s. There are only a few examples of relaxases that show preference for a *cis*-encoded substrate. The MOB_P_ relaxase of transposon Tn*1549* was found to be *cis*-acting (Tsvetkova et al., 2010). Notably, all plasmid-encoded *cis*-acting relaxases have been reported in members of the MOB_Q_ class: TraA of plasmid pRetCFN42d (MOB_Q__2_) (Pérez-Mendoza et al., 2006) and TraA of plasmid pIP501 (MOB_Q__3_) (Arends et al., 2012). Nevertheless, other MOB_Q_ relaxases, such as Nes_pSK41 (Pollet et al., 2016), as well as MobA of plasmids R1162/RSF1010 and pSC101 (Brasch and Meyer, 1986; Derbyshire and Willetts, 1987; Meyer, 2000) worked efficiently *in trans*.

The MOB_Q__4_ relaxases were also tested for their specificity to act on a non-cognate MOB_Q__4_ *oriT*. The *oriT*s of MOB_Q__41_ and MOB_Q__42_ plasmids differ in 10 nucleotides along their 178bp sequence (Supplementary Figure S1). Mobilization frequencies of *oriT*_MOB_Q__42_ plasmids pRC6 or pRC8 by the MOB_Q__41_ plasmid pRC1 + R64*drd11*, as well as *oriT*_MOB_Q__41_ plasmids pRC5 or pRC7 by the MOB_Q__42_ plasmid pRC2 + R64*drd11*, were similar to that obtained for the cognate systems, varying no more than one log (Figure 4).

To further delimit the *oriT* of MOB_Q__4_ plasmids, the 178bp *oriT* fragments cloned in pRC7 and pRC8 (see Supplementary Figure S1) were subdivided in two portions, one containing *oriT* nucleotides 1–70 (pRC11 and pRC12) and the other containing *oriT* nucleotides 71–178 (pRC9 and pRC10) (Figure 1 and Supplementary Figure S1). Disruption of the 178bp *oriT* region resulted in a drastic loss of conjugation efficiency of the *oriT-*containing plasmid (Figure 4), as previously reported for pIGWZ12 (Zaleski et al., 2015).

The *cis*-acting preference of the MOB_Q__4_ relaxases shown here is an example of biological orthogonality (de Lorenzo, 2011), that is, a mechanism to avoid interference. It implies that when two MOB_Q__4_ plasmids are present in the same cell, the contribution of *oriT* cross-recognition by the heterologous MOB_Q__4_ relaxase to plasmid transfer is not substantial. This feature could be essential to guarantee their efficient transfer, given the fact that both types of MOB_Q__4_ plasmids use the same repertoire of conjugative helpers and share the same hosts.

## Conclusion

MOB_Q__41_ and MOB_Q__42_ plasmids are able to coexist and spread in the *E. coli* population without affecting each other largely. They disseminate through bacterial conjugation, aided specially by MPF_I_ conjugative plasmids, but neither of the MOB_Q__4_ plasmids dominates the horizontal transfer process. Co-residence of MOB_Q__41_ and MOB_Q__42_ plasmids in the same host neither hindered nor boosted considerably their respective mobilization frequencies. Since both plasmids (MOB_Q__41_ and MOB_Q__42_) have a narrow host-range (they circulate among enterobacteria), their coexistence in natural environments is likely. In such ecological setting, specific discrimination among their highly similar *oriT* sequences would be guaranteed by the preferential *cis* activity of the MOB_Q__4_ relaxase. Such strategy would be biologically relevant in a scenario of co-residence of non-divergent elements to favor self-dissemination.

## Data Availability Statement

All datasets generated for this study are included in the article/Supplementary  Material.

## Author Contributions

MG-B and FC conceived the study and designed the experiments. RC-L, AC, and MG-B performed the experiments. RC-L, AC, MG-B, and FC interpreted the data. MG-B and FC wrote the manuscript.

## Conflict of Interest

The authors declare that the research was conducted in the absence of any commercial or financial relationships that could be construed as a potential conflict of interest.

## References

[B1] Achtman M. Willetts N. Clark A. J. (1971). Beginning a genetic analysis of conjugational transfer determined by the F factor in *Escherichia coli* by isolation and characterization of transfer-deficient mutants. *J. Bacteriol.* 106 529–538. 4929865 10.1128/jb.106.2.529-538.1971 PMC285127

[B2] Altschul S. F. Madden T. L. Schäffer A. A. Zhang J. Zhang Z. Miller W. (1997). Gapped BLAST and PSI-BLAST: a new generation of protein database search programs. *Nucleic Acids Res.* 25 3389–3402. 10.1093/nar/25.17.3389 9254694 PMC146917

[B3] Alvarado A. Garcillán-Barcia M. P. de la Cruz F. (2012). A degenerate primer MOB typing (DPMT) method to classify gamma-proteobacterial plasmids in clinical and environmental settings. *PLoS One* 7:e40438. 10.1371/journal.pone.0040438 22792321 PMC3394729

[B4] Arends K. Schiwon K. Sakinc T. Hübner J. Grohmann E. (2012). Green fluorescent protein-labeled monitoring tool to quantify conjugative plasmid transfer between gram-positive and gram-negative bacteria. *Appl. Environ. Microbiol.* 78 895–899. 10.1128/AEM.05578-5511 22138997 PMC3264103

[B5] Beatson S. A. Ben Zakour N. L. Totsika M. Forde B. M. Watts R. E. Mabbett A. N. (2015). Molecular analysis of asymptomatic bacteriuria *Escherichia coli* strain VR50 reveals adaptation to the urinary tract by gene acquisition. *Infect. Immun.* 83 1749–1764. 10.1128/IAI.02810-2814 25667270 PMC4399054

[B6] Bleicher A. Schöfl G. Rodicio M. D. R. Saluz H. P. (2013). The plasmidome of a *Salmonella enterica* serovar derby isolated from pork meat. *Plasmid* 69 202–210. 10.1016/j.plasmid.2013.01.001 23333216

[B7] Brasch M. A. Meyer R. J. (1986). Genetic organization of plasmid R1162 DNA involved in conjugative mobilization. *J. Bacteriol.* 167 703–710. 10.1128/jb.167.2.703-710.1986 3525520 PMC212946

[B8] Brolund A. Franzén O. Melefors O. Tegmark-Wisell K. Sandegren L. (2013). Plasmidome-analysis of ESBL-producing *escherichia coli* using conventional typing and high-throughput sequencing. *PLoS One* 8:e65793. 10.1371/journal.pone.0065793 23785449 PMC3681856

[B9] Cabezón E. Lanka E. de la Cruz F. (1994). Requirements for mobilization of plasmids RSF1010 and ColE1 by the IncW plasmid R388: trwB and RP4 traG are interchangeable. *J. Bacteriol.* 176 4455–4458. 10.1128/jb.176.14.4455-4458.1994 8021231 PMC205661

[B10] Cabezón E. Sastre J. I. de la Cruz F. (1997). Genetic evidence of a coupling role for the TraG protein family in bacterial conjugation. *Mol. Gen. Genet.* 254 400–406. 10.1007/s004380050432 9180693

[B11] Calva E. Silva C. Zaidi M. B. Sanchez-Flores A. Estrada K. Silva G. G. Z. (2015). Complete genome sequencing of a multidrug-resistant and human-invasive *Salmonella enterica* serovar typhimurium strain of the emerging sequence type 213 genotype. *Genome Announc.* 3:e663-15. 10.1128/genomeA.00663-615 26089426 PMC4472903

[B12] Capella-Gutiérrez S. Silla-Martínez J. M. Gabaldón T. (2009). trimAl: a tool for automated alignment trimming in large-scale phylogenetic analyses. *Bioinformatics* 25 1972–1973. 10.1093/bioinformatics/btp348 19505945 PMC2712344

[B13] Carattoli A. Zankari E. García-Fernández A. Voldby Larsen M. Lund O. Villa L. (2014). In silico detection and typing of plasmids using plasmidfinder and plasmid multilocus sequence typing. *Antimicrob. Agents Chemother.* 58 3895–3903. 10.1128/AAC.02412-14 24777092 PMC4068535

[B14] Cascales E. Atmakuri K. Liu Z. Binns A. N. Christie P. J. (2005). *Agrobacterium tumefaciens* oncogenic suppressors inhibit T-DNA and VirE2 protein substrate binding to the VirD4 coupling protein. *Mol. Microbiol.* 58 565–579. 10.1111/j.1365-2958.2005.04852.x 16194240 PMC2749481

[B15] Cascales E. Buchanan S. K. Duché D. Kleanthous C. Lloubès R. Postle K. (2007). Colicin biology. *Microbiol. Mol. Biol. Rev.* 71 158–229. 10.1128/MMBR.00036-36 17347522 PMC1847374

[B16] Darriba D. Taboada G. L. Doallo R. Posada D. (2011). ProtTest 3: fast selection of best-fit models of protein evolution. *Bioinformatics* 27 1164–1165. 10.1093/bioinformatics/btr088 21335321 PMC5215816

[B17] Datsenko K. A. Wanner B. L. (2000). One-step inactivation of chromosomal genes in *Escherichia coli* K-12 using PCR products. *Proc. Natl. Acad. Sci. U.S.A.* 97 6640–6645. 10.1073/pnas.120163297 10829079 PMC18686

[B18] de la Cruz F. Frost L. S. Meyer R. J. Zechner E. L. (2010). Conjugative DNA metabolism in gram-negative bacteria. *FEMS Microbiol. Rev.* 34 18–40. 10.1111/j.1574-6976.2009.00195.x 19919603

[B19] de la Cruz F. Grinsted J. (1982). Genetic and molecular characterization of Tn21, a multiple resistance transposon from R100.1. *J. Bacteriol.* 151 222–228. 6282806 10.1128/jb.151.1.222-228.1982 PMC220230

[B20] de Lorenzo V. (2011). Beware of metaphors: chasses and orthogonality in synthetic biology. *Bioeng. Bugs* 2 3–7. 10.4161/bbug.2.1.13388 21636982

[B21] de Toro M. Garcillán-Barcia M. P. De La Cruz F. (2014). Plasmid diversity and adaptation analyzed by massive sequencing of *Escherichia coli* plasmids. *Microbiol. Spectr.* 2 219–235. 10.1128/microbiolspec.PLAS-0031-2014 26104438

[B22] del Campo I. Ruiz R. Cuevas A. Revilla C. Vielva L. de la Cruz F. (2012). Determination of conjugation rates on solid surfaces. *Plasmid* 67 174–182. 10.1016/j.plasmid.2012.01.008 22289895

[B23] DeLong A. Syvanen M. (1991). Trans-acting transposase mutant from Tn5. *Proc. Natl. Acad. Sci. U.S.A.* 88 6072–6076. 10.1073/pnas.88.14.6072 1648727 PMC52024

[B24] Derbyshire K. M. Kramer M. Grindley N. D. (1990). Role of instability in the cis action of the insertion sequence IS903 transposase. *Proc. Natl. Acad. Sci. U.S.A.* 87 4048–4052. 10.1073/pnas.87.11.4048 2161528 PMC54044

[B25] Derbyshire K. M. Willetts N. S. (1987). Mobilization of the non-conjugative plasmid RSF1010: a genetic analysis of its origin of transfer. *Mol. Gen. Genet.* 206 154–160. 10.1007/bf00326551 3033437

[B26] Dunlop M. J. Cox R. S. Levine J. H. Murray R. M. Elowitz M. B. (2008). Regulatory activity revealed by dynamic correlations in gene expression noise. *Nat. Genet.* 40 1493–1498. 10.1038/ng.281 19029898 PMC2829635

[B27] Dyall-Smith M. L. Liu Y. Billman-Jacobe H. (2017). Genome sequence of an australian monophasic *Salmonella enterica* subsp. enterica typhimurium Isolate (TW-Stm6) carrying a large plasmid with multiple antimicrobial resistance genes. *Genome Announc.* 5 e793–17. 10.1128/genomeA.00793-717 PMC5604773 28818900

[B28] Edgar R. C. (2004). MUSCLE: multiple sequence alignment with high accuracy and high throughput. *Nucleic Acids Res.* 32 1792–1797. 10.1093/nar/gkh340 15034147 PMC390337

[B29] Edwards J. S. Betts L. Frazier M. L. Pollet R. M. Kwong S. M. Walton W. G. (2013). Molecular basis of antibiotic multiresistance transfer in *Staphylococcus aureus*. *Proc. Natl. Acad. Sci. U.S.A.* 110 2804–2809. 10.1073/pnas.1219701110 23359708 PMC3581901

[B30] Fernandez-Lopez R. Redondo S. Garcillan-Barcia M. P. de la Cruz F. (2017). Towards a taxonomy of conjugative plasmids. *Curr. Opin. Microbiol.* 38 106–113. 10.1016/j.mib.2017.05.005 28586714

[B31] Finn R. D. Coggill P. Eberhardt R. Y. Eddy S. R. Mistry J. Mitchell A. L. (2016). The Pfam protein families database: towards a more sustainable future. *Nucleic Acids Res.* 44 D279–D285. 10.1093/nar/gkv1344 26673716 PMC4702930

[B32] Francia M. V. Varsaki A. Garcillán-Barcia M. P. Latorre A. Drainas C. de la Cruz F. (2004). A classification scheme for mobilization regions of bacterial plasmids. *FEMS Microbiol. Rev.* 28 79–100. 10.1016/j.femsre.2003.09.001 14975531

[B33] Fratamico P. M. Yan X. Caprioli A. Esposito G. Needleman D. S. Pepe T. (2011). The complete DNA sequence and analysis of the virulence plasmid and of five additional plasmids carried by shiga toxin-producing *Escherichia coli* O26:H11 strain H30. *Int. J. Med. Microbiol.* 301 192–203. 10.1016/j.ijmm.2010.09.002 21212019

[B34] Fricke W. F. Wright M. S. Lindell A. H. Harkins D. M. Baker-Austin C. Ravel J. (2008). Insights into the environmental resistance gene pool from the genome sequence of the multidrug-resistant environmental isolate *Escherichia coli* SMS-3-5. *J. Bacteriol.* 190 6779–6794. 10.1128/JB.00661-668 18708504 PMC2566207

[B35] Furuya N. Nisioka T. Komano T. (1991). Nucleotide sequence and functions of the oriT operon in IncI1 plasmid R64. *J. Bacteriol.* 173 2231–2237. 10.1128/jb.173.7.2231-2237.1991 1848841 PMC207772

[B36] Gama J. A. Zilhão R. Dionisio F. (2017a). Co-resident plasmids travel together. *Plasmid* 93 24–29. 10.1016/j.plasmid.2017.08.004 28842131

[B37] Gama J. A. Zilhão R. Dionisio F. (2017b). Conjugation efficiency depends on intra and intercellular interactions between distinct plasmids: plasmids promote the immigration of other plasmids but repress co-colonizing plasmids. *Plasmid* 93 6–16. 10.1016/j.plasmid.2017.08.003 28842132

[B38] Gama J. A. Zilhão R. Dionisio F. (2017c). Multiple plasmid interference - pledging allegiance to my enemy’s enemy. *Plasmid* 93 17–23. 10.1016/j.plasmid.2017.08.002 28842133

[B39] Gama J. A. Zilhão R. Dionisio F. (2018). Impact of plasmid interactions with the chromosome and other plasmids on the spread of antibiotic resistance. *Plasmid* 99 82–88. 10.1016/j.plasmid.2018.09.009 30240700

[B40] Garcillán-Barcia M. P. Cuartas Lanza R. Cuevas A. de la Cruz F. (2019). Comparative analysis of MOBQ4 plasmids demonstrates that MOBQ is a cis-acting enriched relaxase protein family. *bioRxiv* org/10.1101/726927[Preprint].

[B41] Garcillán-Barcia M. P. de la Cruz F. (2013). Ordering the bestiary of genetic elements transmissible by conjugation. *Mob. Genet. Elements* 3:e24263. 10.4161/mge.24263 23734300 PMC3661145

[B42] Garcillán-Barcia M. P. Francia M. V. de la Cruz F. (2009). The diversity of conjugative relaxases and its application in plasmid classification. *FEMS Microbiol. Rev.* 33 657–687. 10.1111/j.1574-6976.2009.00168.x 19396961

[B43] Garcillán-Barcia M. P. Ruiz del Castillo B. Alvarado A. de la Cruz F. Martínez-Martínez L. (2015). Degenerate primer MOB typing of multiresistant clinical isolates of *E. coli* uncovers new plasmid backbones. *Plasmid* 77 17–27. 10.1016/j.plasmid.2014.11.003 25463772

[B44] Getino M. de la Cruz F. (2018). Natural and artificial strategies to control the conjugative transmission of plasmids. *Microbiol. Spectr* 6 1–25. 10.1128/microbiolspec.MTBP-0015-2016 29327679 PMC11633558

[B45] Getino M. Palencia-Gándara C. Garcillán-Barcia M. P. de la Cruz F. (2017). PifC and osa, plasmid weapons against rival conjugative coupling proteins. *Front. Microbiol.* 8:2260. 10.3389/fmicb.2017.02260 29201021 PMC5696584

[B46] Gibson D. G. Young L. Chuang R.-Y. Venter J. C. Hutchison C. A. Smith H. O. (2009). Enzymatic assembly of DNA molecules up to several hundred kilobases. *Nat. Methods* 6 343–345. 10.1038/nmeth.1318 19363495

[B47] Gibson M. K. Forsberg K. J. Dantas G. (2015). Improved annotation of antibiotic resistance determinants reveals microbial resistomes cluster by ecology. *ISME J.* 9 207–216. 10.1038/ismej.2014.106 25003965 PMC4274418

[B48] Grant S. G. Jessee J. Bloom F. R. Hanahan D. (1990). Differential plasmid rescue from transgenic mouse DNAs into *Escherichia coli* methylation-restriction mutants. *Proc. Natl. Acad. Sci. U.S.A.* 87 4645–4649. 10.1073/pnas.87.12.4645 2162051 PMC54173

[B49] Guglielmini J. Néron B. Abby S. S. Garcillán-Barcia M. P. de la Cruz F. Rocha E. P. C. (2014). Key components of the eight classes of type IV secretion systems involved in bacterial conjugation or protein secretion. *Nucleic Acids Res.* 42 5715–5727. 10.1093/nar/gku194 24623814 PMC4027160

[B50] Guindon S. Gascuel O. (2003). A simple, fast, and accurate algorithm to estimate large phylogenies by maximum likelihood. *Syst. Biol.* 52 696–704. 10.1080/10635150390235520 14530136

[B51] Guiney D. G. Deiss C. Simnad V. Yee L. Pansegrau W. Lanka E. (1989). Mutagenesis of the Tra1 core region of RK2 by using Tn5: identification of plasmid-specific transfer genes. *J. Bacteriol.* 171 4100–4103. 10.1128/jb.171.7.4100-4103.1989 2544570 PMC210173

[B52] Hiraga S. Sugiyama T. Itoh T. (1994). Comparative analysis of the replicon regions of eleven ColE2-related plasmids. *J. Bacteriol.* 176 7233–7243. 10.1128/jb.176.23.7233-7243.1994 7525540 PMC197111

[B53] Holt K. E. Thieu Nga T. V. Thanh D. P. Vinh H. Kim D. W. Vu Tra M. P. (2013). Tracking the establishment of local endemic populations of an emergent enteric pathogen. *Proc. Natl. Acad. Sci. U.S.A.* 110 17522–17527. 10.1073/pnas.1308632110 24082120 PMC3808646

[B54] Horii T. Itoh T. (1988). Replication of ColE2 and ColE3 plasmids: the regions sufficient for autonomous replication. *Mol. Gen. Genet.* 212 225–231. 10.1007/bf00334689 2841566

[B55] Itoh T. Horii T. (1989). Replication of ColE2 and ColE3 plasmids: in vitro replication dependent on plasmid-coded proteins. *Mol. Gen. Genet.* 219 249–255. 10.1007/bf00261184 2693943

[B56] Kelley L. A. Mezulis S. Yates C. M. Wass M. N. Sternberg M. J. E. (2015). The Phyre2 web portal for protein modeling, prediction and analysis. *Nat. Protoc.* 10 845–858. 10.1038/nprot.2015.053 25950237 PMC5298202

[B57] Kido M. Yasueda H. Itoh T. (1991). Identification of a plasmid-coded protein required for initiation of ColE2 DNA replication. *Nucleic Acids Res.* 19 2875–2880. 10.1093/nar/19.11.2875 1829159 PMC328245

[B58] Lanza V. F. de Toro M. Garcillán-Barcia M. P. Mora A. Blanco J. Coque T. M. (2014). Plasmid flux in *Escherichia coli* ST131 sublineages, analyzed by plasmid constellation network (PLACNET), a new method for plasmid reconstruction from whole genome sequences. *PLoS Genet.* 10:e1004766. 10.1371/journal.pgen.1004766 25522143 PMC4270462

[B59] Liu C. Zheng H. Yang M. Xu Z. Wang X. Wei L. (2015). Genome analysis and in vivo virulence of porcine extraintestinal pathogenic *Escherichia coli* strain PCN033. *BMC Genomics* 16:717. 10.1186/s12864-015-1890-1899 26391348 PMC4578781

[B60] Lorenzo-Díaz F. Fernández-López C. Garcillán-Barcia M. P. Espinosa M. (2014). Bringing them together: plasmid pMV158 rolling circle replication and conjugation under an evolutionary perspective. *Plasmid* 74 15–31. 10.1016/j.plasmid.2014.05.004 24942190 PMC7103276

[B61] Maindola P. Raina R. Goyal P. Atmakuri K. Ojha A. Gupta S. (2014). Multiple enzymatic activities of ParB/Srx superfamily mediate sexual conflict among conjugative plasmids. *Nat. Commun.* 5:5322. 10.1038/ncomms6322 25358815 PMC4241021

[B62] Meyer R. (2000). Identification of the mob genes of plasmid pSC101 and characterization of a hybrid pSC101-R1162 system for conjugal mobilization. *J. Bacteriol.* 182 4875–4881. 10.1128/jb.182.17.4875-4881.2000 10940031 PMC111367

[B63] Meyer R. (2009). Replication and conjugative mobilization of broad host-range IncQ plasmids. *Plasmid* 62 57–70. 10.1016/j.plasmid.2009.05.001 19465049 PMC2752045

[B64] Moncalián G. Grandoso G. Llosa M. de la Cruz F. (1997). oriT-processing and regulatory roles of TrwA protein in plasmid R388 conjugation. *J. Mol. Biol.* 270 188–200. 10.1006/jmbi.1997.1082 9236121

[B65] Monzingo A. F. Ozburn A. Xia S. Meyer R. J. Robertus J. D. (2007). The structure of the minimal relaxase domain of MobA at 2.1 a resolution. *J. Mol. Biol.* 366 165–178. 10.1016/j.jmb.2006.11.031 17157875 PMC1894915

[B66] Morisato D. Way J. C. Kim H. J. Kleckner N. (1983). Tn10 transposase acts preferentially on nearby transposon ends in vivo. *Cell* 32 799–807. 10.1016/0092-8674(83)90066-90061 6299577

[B67] Nomura N. Masai H. Inuzuka M. Miyazaki C. Ohtsubo E. Itoh T. (1991). Identification of eleven single-strand initiation sequences (ssi) for priming of DNA replication in the F, R6K, R100 and ColE2 plasmids. *Gene* 108 15–22. 10.1016/0378-1119(91)90482-q 1761225

[B68] Novick R. P. (1987). Plasmid incompatibility. *Microbiol. Rev.* 51 381–395. 3325793 10.1128/mr.51.4.381-395.1987 PMC373122

[B69] Ogura Y. Ooka T. Iguchi A. Toh H. Asadulghani M. Oshima K. (2009). Comparative genomics reveal the mechanism of the parallel evolution of O157 and non-O157 enterohemorrhagic *Escherichia coli*. *Proc. Natl. Acad. Sci. U.S.A.* 106 17939–17944. 10.1073/pnas.0903585106 19815525 PMC2764950

[B70] Oshima K. Toh H. Ogura Y. Sasamoto H. Morita H. Park S.-H. (2008). Complete genome sequence and comparative analysis of the wild-type commensal *Escherichia coli* strain SE11 isolated from a healthy adult. *DNA Res.* 15 375–386. 10.1093/dnares/dsn026 18931093 PMC2608844

[B71] Pérez-Mendoza D. Lucas M. Muñoz S. Herrera-Cervera J. A. Olivares J. de la Cruz F. (2006). The relaxase of the rhizobium etli symbiotic plasmid shows nic site cis-acting preference. *J. Bacteriol.* 188 7488–7499. 10.1128/JB.00701-706 16916896 PMC1636270

[B72] Pollet R. M. Ingle J. D. Hymes J. P. Eakes T. C. Eto K. Y. Kwong S. M. (2016). Processing of nonconjugative resistance plasmids by conjugation nicking enzyme of staphylococci. *J. Bacteriol.* 198 888–897. 10.1128/JB.00832-815 26728193 PMC4772599

[B73] San Millan A. Heilbron K. MacLean R. C. (2014). Positive epistasis between co-infecting plasmids promotes plasmid survival in bacterial populations. *ISME J.* 8 601–612. 10.1038/ismej.2013.182 24152711 PMC3930321

[B74] San Millan A. MacLean R. C. (2017). Fitness costs of plasmids: a limit to plasmid transmission. *Microbiol. Spectr* 5 601–612. 10.1128/microbiolspec.MTBP-0016-2017 28944751 PMC11687550

[B75] Schrödinger L. (2015). *The PyMOL Molecular Graphics System. Version 1*.

[B76] Silva-Rocha R. Martínez-García E. Calles B. Chavarría M. Arce-Rodríguez A. de Las Heras A. (2013). The standard european vector architecture (SEVA): a coherent platform for the analysis and deployment of complex prokaryotic phenotypes. *Nucleic Acids Res.* 41 D666–D675. 10.1093/nar/gks1119 23180763 PMC3531073

[B77] Smillie C. Garcillán-Barcia M. P. Francia M. V. Rocha E. P. C. de la Cruz F. (2010). Mobility of plasmids. *Microbiol. Mol. Biol. Rev.* 74 434–452. 10.1128/MMBR.00020-10 20805406 PMC2937521

[B78] Sørensen A. H. Hansen L. H. Johannesen E. Sørensen S. J. (2003). Conjugative plasmid conferring resistance to olaquindox. *Antimicrob. Agents Chemother.* 47 798–799. 10.1128/aac.47.2.798-799.2003 12543696 PMC151768

[B79] Stamatakis A. (2006). RAxML-VI-HPC: maximum likelihood-based phylogenetic analyses with thousands of taxa and mixed models. *Bioinformatics* 22 2688–2690. 10.1093/bioinformatics/btl446 16928733

[B80] Stephens C. M. Skerker J. M. Sekhon M. S. Arkin A. P. Riley L. W. (2015). Complete genome sequences of four *Escherichia coli* ST95 isolates from bloodstream infections. *Genome Announc* 3:e1241-15. 10.1128/genomeA.01241-1215 26543109 PMC4645194

[B81] Sugiyama T. Itoh T. (1993). Control of ColE2 DNA replication: in vitro binding of the antisense RNA to the Rep mRNA. *Nucleic Acids Res.* 21 5972–5977. 10.1093/nar/21.25.5972 7507236 PMC310483

[B82] Summers D. (1998). Timing, self-control and a sense of direction are the secrets of multicopy plasmid stability. *Mol. Microbiol.* 29 1137–1145. 10.1046/j.1365-2958.1998.01012.x 9767582

[B83] Summers D. K. Sherratt D. J. (1984). Multimerization of high copy number plasmids causes instability: CoIE1 encodes a determinant essential for plasmid monomerization and stability. *Cell* 36 1097–1103. 10.1016/0092-8674(84)90060-90066 6323019

[B84] Summers D. K. Sherratt D. J. (1988). Resolution of ColE1 dimers requires a DNA sequence implicated in the three-dimensional organization of the cer site. *EMBO J.* 7 851–858. 10.1002/j.1460-2075.1988.tb02884.x 3294000 PMC454402

[B85] Takechi S. Yasueda H. Itoh T. (1994). Control of ColE2 plasmid replication: regulation of rep expression by a plasmid-coded antisense RNA. *Mol. Gen. Genet.* 244 49–56. 10.1007/bf00280186 8041361

[B86] Tsvetkova K. Marvaud J.-C. Lambert T. (2010). Analysis of the mobilization functions of the vancomycin resistance transposon Tn1549, a member of a new family of conjugative elements. *J. Bacteriol.* 192 702–713. 10.1128/JB.00680-689 19966009 PMC2812457

[B87] van Zyl L. J. Deane S. M. Rawlings D. E. (2003). Analysis of the mobilization region of the broad-host-range IncQ-like plasmid pTC-F14 and its ability to interact with a related plasmid, pTF-FC2. *J. Bacteriol.* 185 6104–6111. 10.1128/jb.185.20.6104-6111.2003 14526022 PMC225039

[B88] Varsaki A. Moncalián G. Garcillán-Barcia M. del P. Drainas C. de la Cruz F. (2009). Analysis of ColE1 MbeC unveils an extended ribbon-helix-helix family of nicking accessory proteins. *J. Bacteriol.* 191 1446–1455. 10.1128/JB.01342-1348 19114496 PMC2648203

[B89] Yagura M. Itoh T. (2006). The rep protein binding elements of the plasmid ColE2-P9 replication origin. *Biochem. Biophys. Res. Commun.* 345 872–877. 10.1016/j.bbrc.2006.04.168 16707111

[B90] Yagura M. Nishio S.-Y. Kurozumi H. Wang C.-F. Itoh T. (2006). Anatomy of the replication origin of plasmid ColE2-P9. *J. Bacteriol.* 188 999–1010. 10.1128/JB.188.3.999-1010.2006 16428404 PMC1347323

[B91] Yasueda H. Horii T. Itoh T. (1989). Structural and functional organization of ColE2 and ColE3 replicons. *Mol. Gen. Genet.* 215 209–216. 10.1007/bf00339719 2651878

[B92] Yasueda H. Takechi S. Sugiyama T. Itoh T. (1994). Control of ColE2 plasmid replication: negative regulation of the expression of the plasmid-specified initiator protein, Rep, at a posttranscriptional step. *Mol. Gen. Genet.* 244 41–48. 10.1007/bf00280185 8041360

[B93] Zaleski P. Wawrzyniak P. Sobolewska A. Łukasiewicz N. Baran P. Romańczuk K. (2015). pIGWZ12–A cryptic plasmid with a modular structure. *Plasmid* 79 37–47. 10.1016/j.plasmid.2015.04.001 25889268

[B94] Zaleski P. Wawrzyniak P. Sobolewska A. Mikiewicz D. Wojtowicz-Krawiec A. Chojnacka-Puchta L. (2012). New cloning and expression vector derived from *Escherichia coli* plasmid pIGWZ12; a potential vector for a two-plasmid expression system. *Plasmid* 67 264–271. 10.1016/j.plasmid.2011.12.011 22230664

[B95] Zaleski P. Wolinowska R. Strzezek K. Lakomy A. Plucienniczak A. (2006). The complete sequence and segregational stability analysis of a new cryptic plasmid pIGWZ12 from a clinical strain of *Escherichia coli*. *Plasmid* 56 228–232. 10.1016/j.plasmid.2006.05.004 16828160

