# Supplementary material for: Cis-Acting Relaxases Guarantee Independent Mobilization of MOBQ4 Plasmids
Source: Front Microbiol. 2019 Nov 8;10:2557. doi: 10.3389/fmicb.2019.02557 (PMC6856555; doi:10.3389/fmicb.2019.02557)
Supplement: Supplementary file 1 [file Data_Sheet_1.PDF]

Supplementary Table S1. Conjugative plasmids used as helpers in this work.

Plasmid	Description (MPF type)	GenBank Acc. No.	Reference
pRL443	Kanamycin-sensitive derivative of the IncP1α prototype RP4 (MPF$_T$)	NC_001621.1	(1)
R751	IncP1β prototype (MPF$_T$)	NC_001735.4	(2)
R388	IncW prototype (MPF$_T$)	BR000038.1	(3)
pKM101	Deletion derivative of the IncN prototype R46 (MPF$_T$)	NC_003292.1	(4)
R6K*drd1*	Derepressed mutant of the IncX2 prototype R6K (MPF$_T$)	LT827129.1	(5)
pOLA52	IncX1 plasmid (MPF$_T$)	NC_010378.1	(6)
R64*drd11*	Derepressed mutant of the IncI1α prototype R64 (MPF$_I$)	NC_005014.1	(7)
pCTX-M3	IncL/M plasmid (MPF$_I$)	NC_004464.2	(8)
pOX38	Deletion derivative of the IncFI prototype F plasmid (MPF$_F$)	NC_002483.1	(9)
R100-1	Derepressed mutant of the IncFII prototype R100 (MPF$_F$)	NC_002134.1	(10)
drR27	Derepressed mutant of the IncHI1 prototype R27 (MPF$_F$)	NC_002305.1	(11)

Supplementary Table S2. List and main features of MOB_{Q4} plasmids.

Plasmid	GenBank Acc. No.	Plasmid size (bp)	Phenotype traits	Relaxase Acc. No.	MOB_{Q4} clade	Relaxase accessory protein (MobC) Acc. No.	Replication protein pfam	Host	Reference
pIGWZ12	NC_010885.1	4072	-	YP_001966 167.1	1	n. a. (coordinates 2670 – 2419)	PF01051	*E. coli*	(12)
pSE11-6	NC_011411.1	4082	-	WP_001029 343.1	1	YP_00229108 6.1	PF01051	*E. coli*	(13)
pSD4.0	NC_019134.1	4060	-	YP_006957 902.1	1	AFU91603.1	PF01051	*Salmonella enterica*	(14)
pEC08-4	JX238443.1	4082 (partial)	-	AGI05103.1	1	n. a. (coordinates 3296 – 3547)	PF01051	*E. coli*	(15)
pSMS35_4	NC_010486.1	4074	-	WP_001029 346.1	1	YP_00173988 5.1	PF01051	*E. coli*	(16)
pEC29-1	NG_041590.1	4082 (partial)	-	WP_021545 144.1	1	n. a. (coordinates 3296 – 3547)	PF01051	*E. coli*	(15)
pEC299-2	JX238458.1	4088 (partial)	-	AGI05139.1	1	YP_00909050 3.1	PF01051	*E. coli*	(15)
pEC08-3	NG_041585.1	4071 (partial)	-	WP_032495 499.1	1	n. a. (coordinates 3286 – 3537)	PF01051	*E. coli*	(15)

pEC147-4	NG_041598.1	4071 (partial)	-	WP_001029354.1	1	n. a. (coordinates 3286 – 3537)	PF01051	*E. coli*	(15)
pE2022_4	KT693143.1	4082	-	AMP34686.1	1	AMP34683.1	PF01051	*E. coli*	(17)
pFV9873_1	KT693144.1	4081	-	AMP34690.1	1	AMP34689.1	PF01051	*E. coli*	(17)
pE35BA_1	KT693145.1	4130	-	AMP34693.1	1	AMP34692.1	PF01051	*E. coli*	(17)
pEC958B	NZ_HG941720.1	4080	-	WP_001029344.1	1	n.a. (coordinates 3926-37)	PF01051	*E. coli*	(18)
p4_TW-Stm6	NZ_CP019648.1	4083	-	WP_050947461.1	1	WP_000091746.1	PF01051	*Salmonella enterica*	(19)
pEC881_5	NZ_CP019024.1	4063	-	WP_001029354.1	1	WP_000091746.1	PF01051	*E. coli*	
pECAZ153_4	NZ_CP018996.1	4073	-	WP_001029354.1	1	WP_000091746.1	PF01051	*E. coli*	
pEC867_3	NZ_CP018980.1	4074	-	WP_023146018.1	1	WP_000091747.1	PF01051	*E. coli*	
pEC545_4	NZ_CP018972.1	4073	-	WP_001029354.1	1	WP_000091746.1	PF01051	*E. coli*	
pEC542_4	NZ_CP018966.1	4087	-	WP_001029343.1	1	WP_000091745.1	PF01051	*E. coli*	

pEC224_6	NZ_CP018942.1	4063	-	WP_001029354.1	1	WP_000091746.1	PF01051	*E. coli*	
pEC224_7	NZ_CP018941.1	4062	-	WP_078207086.1	1	WP_000091746.1	PF01051	*E. coli*	
pMRSN346647_4.1	NZ_CP018208.1	4074	-	WP_016245161.1	1	WP_000091748.1	PF01051	*E. coli*	
pC06114_7	NZ_CP016041.1	4071	-	WP_021526521.1	1	WP_000091746.1	PF01051	*E. coli*	
pEC732_4	NZ_CP015142.1	4072	-	WP_001029353.1	1	WP_000091747.1	PF01051	*E. coli*	
pSF-468-4	NZ_CP012629.1	4072	-	WP_001029353.1	1	WP_001322464.1	PF01051	*E. coli*	(20)
pYU39_5.1	NZ_CP011432.1	5093	-	WP_032347464	1	WP_032347463.1	PF01051	*Salmonella enterica*	(21)
pVR50F	NZ_CP011140.1	4075	-	WP_001029350.1	1	WP_000091749.1	PF01051	*E. coli*	(22)
pEC648_4	NZ_CP008718.1	4073	-	WP_001029349.1	1	WP_000091744.1	PF01051	*E. coli*	

p2PCN033	NZ_CP006634.1	4086	-	WP_001029 343.1	1	WP_00009174 5.1	PF01051	*E. coli*	(23)
pMG828-2	NC_008487.1	4091	-	YP_794130. 1	1	n. a. (coordinates 2399 – 2686)	PF01051	*E. coli*	
ColE9-J	NC_011977.1	7577	Colicin E9	YP_002533 53.6.1	2	YP_00253354 2.1	PF03090 + PF08708	*Escherichia coli*	(24)
pColE8	NC_012882.1	6751	Colicin E8	YP_002995 727.1	2	n. a. (coordinates 5732 – 5469)	PF03090 + PF08708	*E. coli*	(25)
pACN001-D	NC_023325.1	6747	Colicin E9	YP_008998 321.1	2	n. a. (coordinates 6656 – 6384)	PF03090 + PF08708	*E. coli*	
pO111_4	NC_013367.1	8140	Colicin E2-like	WP_001029 340.1	2	YP_00323782 3.1	PF03090 + PF08708	*E. coli*	(26)
pO26-S4	NC_011228.1	6758	Colicin E2	YP_002221 663.1	2	YP_00222166 2.1	PF03090 + PF08708 *	*E. coli*	(27)
pIGMS5	NC_010883.1	6750	Colicin E2	YP_001966 151.1	2	n. a. (coordinates 123 – 6601)	PF03090 + PF08708	*E. coli*	
pDPT1	NC_022585.1	6774	Colicin E5	YP_008691 080.1	2	YP_00869107 9.1	PF03090 + PF08708 *	*Shigella sonnei*	(28)
unnamed3	NZ_CP014098.1	8141	Colicin E6	WP_061066 729.1	2	WP_06106672 8.1	PF03090 + PF08708	*Shigella sonnei*	

pDPT3	NC_020412.1	8338	Colicin E5, (artificially inserted KmR)	YP_007500 966.1	2	n. a. (coordinates 1870-1607)	PF03090 + PF08708	*Shigella sonnei*	(28)
pXF5823	NG_035409.1	5823 (partial)	-	YP_009076 807.1	outgroup	-	PF01051	*Xylella fastidiosa*	(29)

n. a.: not annotated in the GenBank file. In parentheses the coordinates of the MobC CDS at the corresponding accession number, manually searched in this work.

*: not annotated in the corresponding GenBank Acc. No. Coordinates 5669 – 6559 for pO26-S4 and 5736 – 6626 for pDPT1.

Table S3. Mobilization frequencies of MOB_{Q4} plasmids by different MPF type helpers.

	MPF type of the helper plasmid										
	MPF_T						MPF_I		MPF_F		
Mobilizable plasmid	RP4	R751	R388	pKM101	R6K*drd1*	pOLA52	R64*drd11*	pCTX-M3	pOX38	R100-1	R27*dr*
pRC1 (MOB_{Q41})	$1.6x10^{-2}$ ($1x10^{-2}$ – $2.7x10^{-2}$)	$1.7x10^{-4}$ ($2.1x10^{-5}$ – $7.6x10^{-5}$)	$<1x10^{-7}$	$<1x10^{-7}$	$1.1x10^{-6}$ ($2.7x10^{-7}$ - $4.3 x10^{-6}$)	$<1x10^{-7}$	$5.1x10^{-1}$ ($3.9x10^{-1}$ – $6.7x10^{-1}$)	n.d.	$<1x10^{-7}$	n.d.	$<1x10^{-7}$
pRC2 (MOB_{Q42})	$1.2x10^{-1}$ ($8.4x10^{-2}$ - $1.8x10^{-1}$)	$7.8x10^{-5}$ ($6.3x10^{-5}$ - $9.7x10^{-5}$)	$<1x10^{-7}$	$<1x10^{-7}$	$1.1x10^{-4}$ ($7.8x10^{-5}$ - $1.6 x10^{-4}$)	$<1x10^{-7}$	1.2 ($4.6x10^{-1}$ – 3.2)	$1.0x10^{-3}$ ($4.5x10^{-4}$ – $2.4x10^{-3}$)	$<1x10^{-7}$	$<1x10^{-7}$	$<1x10^{-7}$

n.d.: Not determined due to the lack of an appropriate AbR marker.

References:

1. **Elhai J, Vepritskiy A, Muro-Pastor AM, Flores E, Wolk CP.** 1997. Reduction of conjugal transfer efficiency by three restriction activities of Anabaena sp. strain PCC 7120. J Bacteriol **179:**1998-2005.
2. **Thorsted PB, Macartney DP, Akhtar P, Haines AS, Ali N, Davidson P, Stafford T, Pocklington MJ, Pansegrau W, Wilkins BM, Lanka E, Thomas CM.** 1998. Complete sequence of the IncPbeta plasmid R751: implications for evolution and organisation of the IncP backbone. J Mol Biol **282:**969-990.
3. **Revilla C, Garcillan-Barcia MP, Fernandez-Lopez R, Thomson NR, Sanders M, Cheung M, Thomas CM, de la Cruz F.** 2008. Different pathways to acquiring resistance genes illustrated by the recent evolution of IncW plasmids. Antimicrob Agents Chemother **52:**1472-1480.
4. **Brown AM, Coupland GM, Willetts NS.** 1984. Characterization of IS46, an insertion sequence found on two IncN plasmids. J Bacteriol **159:**472-481.
5. **Avila P, Nunez B, de la Cruz F.** 1996. Plasmid R6K contains two functional oriTs which can assemble simultaneously in relaxosomes in vivo. J Mol Biol **261:**135-143.
6. **Norman A, Hansen LH, She Q, Sorensen SJ.** 2008. Nucleotide sequence of pOLA52: a conjugative IncX1 plasmid from Escherichia coli which enables biofilm formation and multidrug efflux. Plasmid **60:**59-74.

7. **Sampei G, Furuya N, Tachibana K, Saitou Y, Suzuki T, Mizobuchi K, Komano T.** 2010. Complete genome sequence of the incompatibility group I1 plasmid R64. Plasmid **64:**92-103.

8. **Golebiewski M, Kern-Zdanowicz I, Zienkiewicz M, Adamczyk M, Zylinska J, Baraniak A, Gniadkowski M, Bardowski J, Ceglowski P.** 2007. Complete nucleotide sequence of the pCTX-M3 plasmid and its involvement in spread of the extended-spectrum beta-lactamase gene blaCTX-M-3. Antimicrob Agents Chemother **51:**3789-3795.

9. **Chandler M, Galas DJ.** 1983. IS1-mediated tandem duplication of plasmid pBR322. Dependence on recA and on DNA polymerase I. J Mol Biol **165:**183-190.

10. **Anthony KG, Klimke WA, Manchak J, Frost LS.** 1999. Comparison of proteins involved in pilus synthesis and mating pair stabilization from the related plasmids F and R100-1: insights into the mechanism of conjugation. J Bacteriol **181:**5149-5159.

11. **Gibert M, Juarez A, Madrid C, Balsalobre C.** 2013. New insights in the role of HtdA in the regulation of R27 conjugation. Plasmid **70:**61-68.

12. **Zaleski P, Wolinowska R, Strzezek K, Lakomy A, Plucienniczak A.** 2006. The complete sequence and segregational stability analysis of a new cryptic plasmid pIGWZ12 from a clinical strain of Escherichia coli. Plasmid **56:**228-232.

13. **Oshima K, Toh H, Ogura Y, Sasamoto H, Morita H, Park SH, Ooka T, Iyoda S, Taylor TD, Hayashi T, Itoh K, Hattori M.** 2008. Complete genome sequence and comparative analysis of the wild-type commensal Escherichia coli strain SE11 isolated from a healthy

adult. DNA Res **15:**375-386.

14. **Bleicher A, Schofl G, Rodicio Mdel R, Saluz HP.** 2013. The plasmidome of a Salmonella enterica serovar Derby isolated from pork meat. Plasmid **69:**202-210.

15. **Brolund A, Franzen O, Melefors O, Tegmark-Wisell K, Sandegren L.** 2013. Plasmidome-analysis of ESBL-producing escherichia coli using conventional typing and high-throughput sequencing. PLoS One **8:**e65793.

16. **Fricke WF, Wright MS, Lindell AH, Harkins DM, Baker-Austin C, Ravel J, Stepanauskas R.** 2008. Insights into the environmental resistance gene pool from the genome sequence of the multidrug-resistant environmental isolate Escherichia coli SMS-3-5. J Bacteriol **190:**6779-6794.

17. **Lanza VF, de Toro M, Garcillan-Barcia MP, Mora A, Blanco J, Coque TM, de la Cruz F.** 2014. Plasmid Flux in Escherichia coli ST131 Sublineages, Analyzed by Plasmid Constellation Network (PLACNET), a New Method for Plasmid Reconstruction from Whole Genome Sequences. PLoS Genet **10:**e1004766.

18. **Forde BM, Ben Zakour NL, Stanton-Cook M, Phan MD, Totsika M, Peters KM, Chan KG, Schembri MA, Upton M, Beatson SA.** 2014. The complete genome sequence of Escherichia coli EC958: a high quality reference sequence for the globally disseminated multidrug resistant E. coli O25b:H4-ST131 clone. PLoS One **9:**e104400.

19. **Dyall-Smith ML, Liu Y, Billman-Jacobe H.** 2017. Genome Sequence of an Australian Monophasic Salmonella enterica subsp. enterica

Typhimurium Isolate (TW-Stm6) Carrying a Large Plasmid with Multiple Antimicrobial Resistance Genes. Genome Announc **5**.

20. **Stephens CM, Skerker JM, Sekhon MS, Arkin AP, Riley LW.** 2015. Complete Genome Sequences of Four Escherichia coli ST95 Isolates from Bloodstream Infections. Genome Announc **3**.

21. **Calva E, Silva C, Zaidi MB, Sanchez-Flores A, Estrada K, Silva GG, Soto-Jimenez LM, Wiesner M, Fernandez-Mora M, Edwards RA, Vinuesa P.** 2015. Complete Genome Sequencing of a Multidrug-Resistant and Human-Invasive Salmonella enterica Serovar Typhimurium Strain of the Emerging Sequence Type 213 Genotype. Genome Announc **3**.

22. **Beatson SA, Ben Zakour NL, Totsika M, Forde BM, Watts RE, Mabbett AN, Szubert JM, Sarkar S, Phan MD, Peters KM, Petty NK, Alikhan NF, Sullivan MJ, Gawthorne JA, Stanton-Cook M, Nhu NT, Chong TM, Yin WF, Chan KG, Hancock V, Ussery DW, Ulett GC, Schembri MA.** 2015. Molecular analysis of asymptomatic bacteriuria Escherichia coli strain VR50 reveals adaptation to the urinary tract by gene acquisition. Infect Immun **83:**1749-1764.

23. **Liu C, Zheng H, Yang M, Xu Z, Wang X, Wei L, Tang B, Liu F, Zhang Y, Ding Y, Tang X, Wu B, Johnson TJ, Chen H, Tan C.** 2015. Genome analysis and in vivo virulence of porcine extraintestinal pathogenic Escherichia coli strain PCN033. BMC Genomics **16:**717.

24. **Chak KF, James R.** 1986. Characterization of the ColE9-J plasmid and analysis of its genetic organization. J Gen Microbiol **132:**61-70.

25. **Le Gac M, Doebeli M.** 2010. Environmental viscosity does not affect the evolution of cooperation during experimental evolution of

colicigenic bacteria. Evolution **64:**522-533.

26. **Ogura Y, Ooka T, Iguchi A, Toh H, Asadulghani M, Oshima K, Kodama T, Abe H, Nakayama K, Kurokawa K, Tobe T, Hattori M, Hayashi T.** 2009. Comparative genomics reveal the mechanism of the parallel evolution of O157 and non-O157 enterohemorrhagic Escherichia coli. Proc Natl Acad Sci U S A **106:**17939-17944.

27. **Fratamico PM, Yan X, Caprioli A, Esposito G, Needleman DS, Pepe T, Tozzoli R, Cortesi ML, Morabito S.** 2011. The complete DNA sequence and analysis of the virulence plasmid and of five additional plasmids carried by Shiga toxin-producing Escherichia coli O26:H11 strain H30. Int J Med Microbiol **301:**192-203.

28. **Holt KE, Thieu Nga TV, Thanh DP, Vinh H, Kim DW, Vu Tra MP, Campbell JI, Hoang NV, Vinh NT, Minh PV, Thuy CT, Nga TT, Thompson C, Dung TT, Nhu NT, Vinh PV, Tuyet PT, Phuc HL, Lien NT, Phu BD, Ai NT, Tien NM, Dong N, Parry CM, Hien TT, Farrar JJ, Parkhill J, Dougan G, Thomson NR, Baker S.** 2013. Tracking the establishment of local endemic populations of an emergent enteric pathogen. Proc Natl Acad Sci U S A **110:**17522-17527.

29. **Qin X, Hartung JS.** 2001. Construction of a shuttle vector and transformation of Xylella fastidiosa with plasmid DNA. Curr Microbiol **43:**158-162.
