# Supplementary figures and images for: Cis-Acting Relaxases Guarantee Independent Mobilization of MOBQ4 Plasmids
Source: Front Microbiol. 2019 Nov 8;10:2557. doi: 10.3389/fmicb.2019.02557 (PMC6856555; doi:10.3389/fmicb.2019.02557)

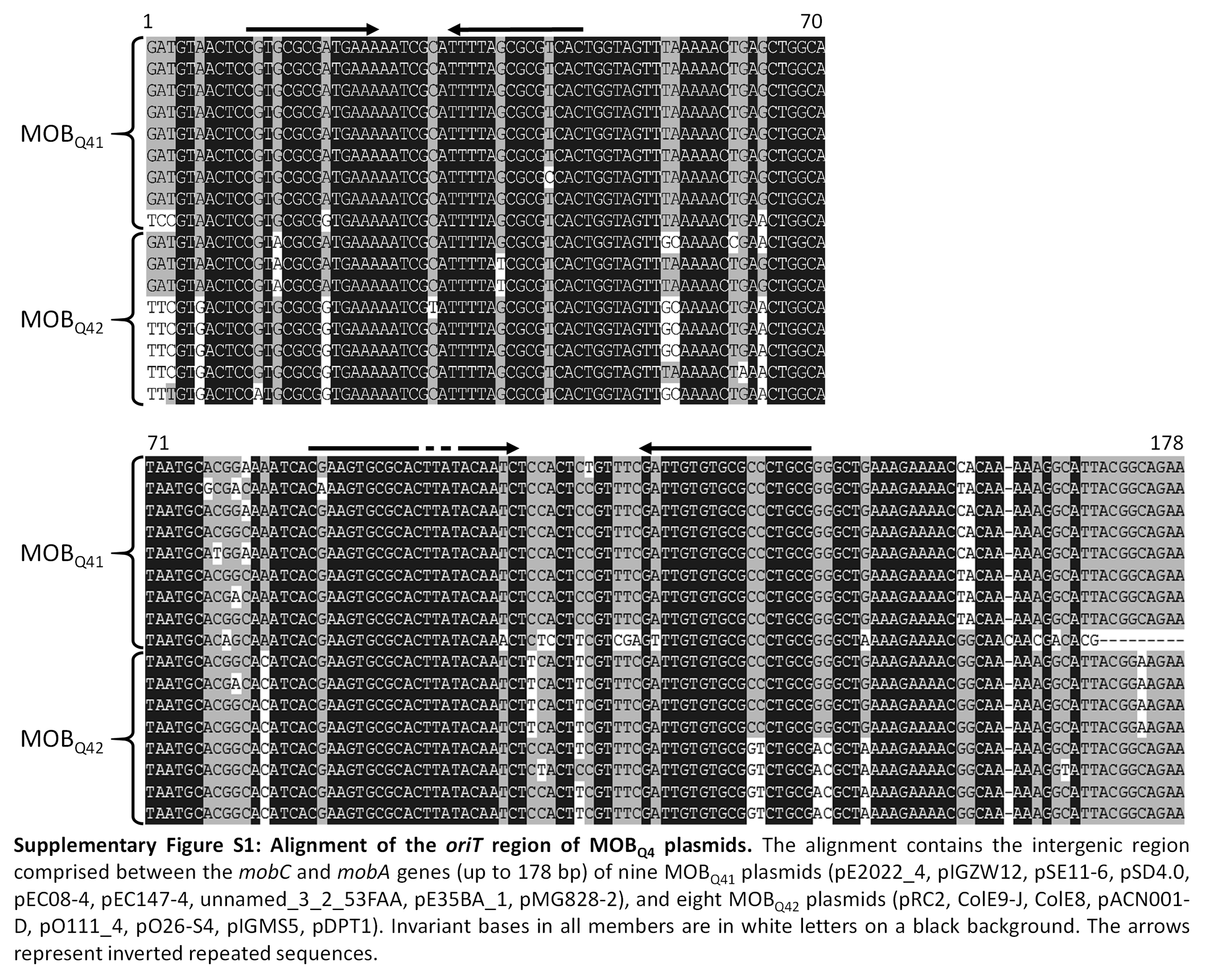

Supplement: Supplementary file 2 [file Image_-1.tif]
